# Development of a Machine-Learning-Based Classifier for the Identification of Head and Body Impacts in Elite Level Australian Rules Football Players

**DOI:** 10.3389/fspor.2021.725245

**Published:** 2021-11-19

**Authors:** Peter Goodin, Andrew J. Gardner, Nasim Dokani, Ben Nizette, Saeed Ahmadizadeh, Suzi Edwards, Grant L. Iverson

**Affiliations:** ^1^School of Medicine, The University of Melbourne, Parkville, VIC, Australia; ^2^HitIQ Ltd., South Melbourne, VIC, Australia; ^3^Priority Research Centre for Stroke and Brain Injury, School of Medicine and Public Health, University of Newcastle, Callaghan, NSW, Australia; ^4^Hunter New England Local Health District Sports Concussion Clinic Research Program, Calvary Mater Hospital, Waratah, NSW, Australia; ^5^Hunter Medical Research Institute, New Lambton Heights, NSW, Australia; ^6^School of Environmental and Life Sciences, The University of Newcastle, Ourimbah, NSW, Australia; ^7^Priority Research Centre for Physical Activity and Nutrition, The University of Newcastle, Callaghan, NSW, Australia; ^8^Department of Physical Medicine and Rehabilitation, Harvard Medical School, Boston, MA, United States; ^9^Department of Physical Medicine and Rehabilitation, Spaulding Rehabilitation Hospital, Charlestown, MA, United States; ^10^Spaulding Research Institute, Charlestown, MA, United States; ^11^Sports Concussion Program, MassGeneral Hospital for Children, Boston, MA, United States; ^12^Home Base, A Red Sox Foundation and Massachusetts General Hospital Program, Charlestown, MA, United States

**Keywords:** Australian football, brain concussion, instrumented mouthguard, kinematics, impacts, machine learning

## Abstract

**Background:** Exposure to thousands of head and body impacts during a career in contact and collision sports may contribute to current or later life issues related to brain health. Wearable technology enables the measurement of impact exposure. The validation of impact detection is required for accurate exposure monitoring. In this study, we present a method of automatic identification (classification) of head and body impacts using an instrumented mouthguard, video-verified impacts, and machine-learning algorithms.

**Methods:** Time series data were collected *via* the Nexus A9 mouthguard from 60 elite level men (mean age = 26.33; SD = 3.79) and four women (mean age = 25.50; SD = 5.91) from the Australian Rules Football players from eight clubs, participating in 119 games during the 2020 season. Ground truth data labeling on the captures used in this machine learning study was performed through the analysis of game footage by two expert video reviewers using SportCode and Catapult Vision. The visual labeling process occurred independently of the mouthguard time series data. True positive captures (captures where the reviewer directly observed contact between the mouthguard wearer and another player, the ball, or the ground) were defined as hits. Spectral and convolutional kernel based features were extracted from time series data. Performances of untuned classification algorithms from scikit-learn in addition to XGBoost were assessed to select the best performing baseline method for tuning.

**Results:** Based on performance, XGBoost was selected as the classifier algorithm for tuning. A total of 13,712 video verified captures were collected and used to train and validate the classifier. True positive detection ranged from 94.67% in the Test set to 100% in the hold out set. True negatives ranged from 95.65 to 96.83% in the test and rest sets, respectively.

**Discussion and conclusion:** This study suggests the potential for high performing impact classification models to be used for Australian Rules Football and highlights the importance of frequencies <150 Hz for the identification of these impacts.

## Introduction

Concussion is a common injury in contact and collision sports (Donaldson et al., 2013; Gardner et al., 2014a,b; Makdissi and Davis, 2016; Dai et al., 2018; Ramkumar et al., 2019). There has been considerable medical interest in improving the identification and management of sport-related concussion (McCrea et al., 2013; McCrory et al., 2017). A number of professional sporting leagues, for example, the Australian Football League (AFL) (Davis et al., 2019a), National Football League (Ellenbogen et al., 2018; Davis et al., 2019a), National Hockey League (Davis et al., 2019a), professional rugby union (Gardner et al., 2018), and the National Rugby League (Davis et al., 2019a), have implemented sideline video surveillance as a strategy for improving the identification of concussion (Davis et al., 2019a,b). This is an important strategy. However, concern has also been raised that it may not only be concussion risk to the health of contact and collision sport athletes, but also the career accumulation of sub concussive impacts that may result in current or future health issues (Gavett et al., 2011; Baugh et al., 2012). Researchers have reported that sub concussive head impacts are associated modest elevations of blood biomarkers over a single practice session of American football (Rubin et al., 2019), and college football players might sustain 1,000 or more sub concussive impacts to the head over the course of a season (Gysland et al., 2011; Bazarian et al., 2014). Cumulative exposure to repetitive head impacts, over time during a single season, might be a risk factor for sustaining a concussion during that season in elite American college football players (Stemper et al., 2018), but cumulative exposure to head impacts was not associated with concussion risk in high school football players (Eckner et al., 2011). Researchers have reported that repetitive head impacts are correlated with changes on experimental brain imaging over the course of a season (Merchant-Borna et al., 2016), and cumulative repetitive head impact exposure is associated with later in life deficits in cognitive functioning and symptoms of depression (Montenigro et al., 2017).

A strategy for evaluating player impact loads as part of an injury prevention program is the use of instrumented technology (Wu et al., 2018; Patton et al., 2020). However, the implementation in the field has been limited by the reliability and validity of such technology (Patton et al., 2020). Using simple peak linear acceleration thresholds to differentiate impacts from normal motion is highly likely to be an insufficient method and is fraught with complex challenges. For example, setting a low magnitude acceleration threshold will increase the likelihood of false positive data, whereas setting a high acceleration threshold will likely result in filtering out some true impacts, and the high acceleration false positives will still remain (Wu et al., 2018). In addition, there are concerns that the majority of the research using sensor-recorded events lack a verification method to confirm the accuracy of the instrumented technology to identify impact loads (Patton et al., 2020). As a result, the absence of a verification method to confirm sensor-recorded events and to remove false positives may be factor in overestimation of head impact exposures (Press and Rowson, 2016; Cortes et al., 2017; Carey et al., 2019; Patton et al., 2020).

Video review, while not infallible and reliant on the skill of the reviewer, has been shown to be a reasonable method for impact detection, with the significant drawback of being labor-intensive (Caswell et al., 2017; Carey et al., 2019; Bailey et al., 2020; Patton et al., 2020). Other detection methods include using filtering algorithms or statistically modeling the impact signature/characteristics (Baugh et al., 2012) to determine whether or not the data is consistent with an impact. This “classification” step, together with the video identification, can be used to evaluate reliability, validity, and accuracy. With the introduction of machine learning based models, high performances for impact identification (>90% accuracy) can be achieved (Baugh et al., 2012). However, these models tend to be trained using single sport data [e.g., American football (Baugh et al., 2012)], which may not be generalizable to other contact or collision sports. The Nexus A9 mouthguard is capable of capturing kinematic data from collisions, such as those that occur in contact sport. To date, there are no statistical models present in the literature to identify impacts in Australian Rules Football. Therefore, the aim of this study was to develop and validate an impact classification method (classifier) for Australian Rules Football, with mouthguard events (captures) recorded using the Nexus A9 mouthguard. Given previous work in this area (Wu et al., 2018; Gabler et al., 2020), we hypothesized it would be possible to develop a machine-learning-based classifier with high performance for delineating hit and non-hit captures.

## Methods

### Study Design

This study was conducted with elite level Australian Football League AFL and Women's AFL (AFLW) players. Consenting participants were provided with custom fit, instrumented mouthguards at the beginning of the season and were requested to wear them during match play. Each team was assigned an account manager, who had the role of distributing the mouthguards to the correct players before the match and then collecting them post-match for cleaning, storage, and uploading of the data from the mouthguard while they were housed in the storage and recharging unit. All matches were televised *via* the league's contracted broadcasters, and footage from the broadcasters were reviewed as part of the video verification process (described in detail below). This study was approved by the University of Newcastle Human Ethics Committee (H-2019-0341).

### Data Collection

Data for the classifier were collected from 64 elite level athletes from eight clubs across 119 matches for which consenting players were participating during the 2020 Australian Football League (AFL) season. There were 60 male AFL players (mean age = 26.33; SD = 3.79) and four female players from the Women's AFL (AFLW; mean age = 25.50; SD = 5.91). A total of 21,348 potential impacts (captures) were generated of which 13,744 were used for training and validation purposes (see Data Preprocessing).

### Mouthguard Specifications

The HitIQ Nexus A9 instrumented mouth guard (HitIQ Pty. Ltd.) used in this study contained three triaxial accelerometers (Analog Devices ADXL372, range: ±200 G, 12-bit) and a gyroscope (Bosch BMG 250, ±2,000 dps range, 16-bit). These were sampled at 3,200 and 800 Hz, respectively. The circuit board and components such as a battery and antenna system were embedded in the mouthguard using a proprietary process. A three-accelerometer array located in the left, central, and right regions of the mouthguard was used to provide an estimate of the angular acceleration independent of the gyroscope and allowed for a crosscheck to remove spurious readings, such as those originating from actions like mouthguard deformation rather than head kinematics. The Nexus A9 mouthguard has been shown to have good concordance with reference sensors in drop tests [LCCC = 0.997 (Stitt et al., 2021)].

### Capture Recording

Recorded mouthguard events (captures) were identified based on thresholding the normed signal from the left linear accelerometer at 10 g's or greater. This magnitude threshold was chosen because below 10 g's has been reported to be indicative of non-impact events (e.g., walking, sitting, etc.) (King et al., 2016). A capture consisted of a lead in the period of 20 ms prior to the 10 g threshold being reached and ended 80 ms after the last trigger event. This allowed for multiple impact events to be recorded in a single capture. The capture was then stored in onboard memory in the mouthguard.

### Data Processing

Due to individual variation within linear accelerometer sampling rates, time series for each axis of the three linear accelerometer sensors were resampled to 3,200 Hz. Gyroscope data were upsampled from 800 to 3,200 Hz. All resampling was carried out using polyphase filtering as present in scipy's resample polyfunction.

Resampled data were triaged to decrease the number of vocalization signals or those consisting of high frequency noise (Wu et al., 2018). The normed signal from the left linear accelerometer was low pass filtered at 300 Hz using a Butterworth second order non-phase corrected filter and subject to a 10 g threshold. Captures that passed the triage were included in the final training/validation data.

### Data Labeling

Ground truth data labeling on the captures used in this machine learning study was performed through analysis of game footage by two expert video reviewers using SportCode (https://www.hudl.com/en_gb/products/sportscode) and Catapult Vision (https://www.catapultsports.com/products/vision). The visual labeling process occurred independently of the mouthguard time series data. Reviewers were provided with video footage (720p, 50 frames per second) from four angles, namely a broadcast view with a tight field of view on the ball, a side view, and footage from behind the goals, to determine if a capture represented a legitimate impact (hit). Time stamps of captures were chronologically synchronized with video footage with start and end times provided by the AFL. Obvious hit events were used to make fine adjustments to the synchronization process (within ± 1 s). Capture events were viewed and labeled according to several predefined labels. Study participants (i.e., those wearing the mouth guard) were identified from their AFL guernsey numbers and from known physical characteristics. Captures where the reviewer directly observed contact between the mouthguard wearer and another player, or the ball, or the ground were labeled as hits. Captures where no contact was observed were given a general label (non-hit) and given a sublabel based on the activity observed—hit, biting, chewing, drinking, mouthguard insertion, mouthguard removal, mouthguard in hand, mouthguard in sock, yelling, no video footage (on sideline), and unknown (if video footage was available, but insufficient to directly observe the event). Quantification of hits that failed to reach the 10 g capture trigger threshold (see section Mouthguard Specifications) was not undertaken.

### Datasets

Data that passed the triage process (13,712 captures) were divided into two sets, a classifier training and a validation set (Set 1) and a separate hold out set (Set 2). Set 1 contained 13,417 captures (1,580 hits, 11,837 non-hits), which were balanced by downsampling the majority class (non-hit) to the minority, selecting captures to be included through pseudorandom sampling using a uniform distribution. The balanced set (3,160 captures) was divided into training (70% of the balanced data), validation (15%), and test (15%) subsets. Set 2 consisted of captures acquired from a single match that were not included in Set 1(Holdout; 57 hits, 238 non-hits).

The validation set was used to estimate the unbiased error of the tuned hyperparameters. Test, rest, and holdout sets were used to examine how the end model would perform given hypothetical scenarios. The test dataset was used as an additional estimate of model performance given reasonably balanced classes. Conversely, the rest subset consisted of non-hit captures that were not included in the training, validation, or test subsets (10,257 non-hit captures). Since our data showed a large imbalance toward nonhits (roughly 10:1), the rest dataset was used to examine the real-world specificity profile of the model. The holdout set was used to examine model performance on unseen data.

### Feature Generation

Features were calculated on signals from all axes of the three linear accelerometers and the gyrometer (12 signals total). Signals were first aligned to cardinal axes using rotational matrices derived from a proprietary calibration process unique for each mouthguard.

Two families of features were generated to capitalize on the shape and spectral characteristics of the signals. Random convolutional kernels were generated (Rahimi and Recht, 2007), with each signal standardized to the signal mean and standard deviation. Three hundred kernels were generated, with the maximum value of the kernel and number of values greater than zero extracted per kernel. A total of 600 features were generated per signal.

Spectral characteristics were examined by calculating the power spectra density of each signal, using scipy's (Oliphant, 2007) implementation of Welch's method (Welch, 1967). Power spectra densities were split into 10-Hz bins, the characteristic value of the bin extracted, and then natural log transformed. The 1,908 power spectra density and 720 convolutional kernel features were then standardized to the mean and standard deviation of the training set.

### Classifier Selection

Selection of a classification algorithm to use for final modeling was achieved by assessing performance of untuned algorithms on the training dataset. All available classification methods present in Scikit-learn (Abraham et al., 2014) were examined. Due to its popularity and performance, the eXtreme gradient boosting (XGBoost) algorithm was also included (Chen and Guestrin, 2016). Default settings for each algorithm were used. Performance was assessed based on the number of hits correctly classified as hits (true positive; TP) and the number of non-hits correctly classified (true negative; TN).

The estimator with the highest TP and TN performance and the least difference between performance metrics in the validation set was chosen for further tuning. The least difference was included as a selection criterion to select a classification algorithm that would be unbiased toward label type.

### Classifier Training and Evaluation

Randomized Search CV was used to train the highest performing estimator, optimizing using Matthew's correlation coefficient. Fifty candidate combinations of parameters were selected using 5-fold cross validation, for a total of 500 fits. The highest performing combination of hyper parameters was used for further performance validation.

Generalizability of classifier performance was assessed using TP and TN metrics and the F1 score on the validation, test, rest, and hold out data. Performance bounds were calculated using bootstrapped 95% confidence intervals, generated across 10,000 shuffles, with data selected pseudorandomly using a uniform distribution.

### Model Interpretation

To assist with model interpretation, including insights into feature importance and the impact of features on individual observation, SHapley Additive exPlanations (SHAP)'s TreeExplainer method was used (Lundberg and Lee, 2017; Lundberg et al., 2020). The validation dataset was used to generate SHAP values.

## Results

### Classifier Selection Analysis

True positive, (TN), and the absolute difference between metrics (TP – TN) for all valid classifier algorithms in SKlearn and XGBoost are presented in Table 1. The mean classifier performance for TPs and TNs was 77.84% (standard deviation = 31.77%) and 89.55% (standard deviation = 11.77%) respectively, with TP values ranging from 0% (Gaussian process classifier, label propagation, label spreading, quadratic discriminant analysis) to 98.04% (passive aggressive classifier, perceptron) and TNs ranging from 47.53% (dummy classifier) to 100% (Gaussian process classifier, label propagation, label spreading, quadratic discriminant analysis). Due to the mixed results between histogram-based gradient boosting (HGB) and XGBoost for our selection criteria, a second comparison was performed on fully tuned models. The results (Table 2) showed no clear superior algorithm between the two. HGB is also an experimental method within sklearn that has not been fully tested. Therefore, we decided to continue with analysis of the XGBoost-based model.

**Table 1:** Untuned algorithm performance for true positive (TP), true negative (TN), and difference between TP, and TN (TP – TN) performance on the training set.

**Algorithm**	**TP (%)**	**TN (%)**	**TP – TN (%)**
Gaussian process classifier	0	100	100
Label propagation	0	100	100
Label spreading	0	100	100
Quadratic discriminant analysis	11.91	90.48	78.57
Bernoulli NB	61.4	83.48	22.07
Passive aggressive classifier	82.03	69.13	−12.9
Perceptron	96.8	84.99	−11.82
Nearest centroid	84.26	75.65	−8.61
Complement NB	84.26	75.65	−8.61
Multinomial NB	93.94	87.13	−6.81
Extra tree classifier	84.43	78.55	−5.87
K neighbors classifier	94.61	89.14	−5.47
Logistic regression	81.56	76.54	−5.02
Bagging classifier	56.15	52.01	−4.14
Ridge classifier CV	82.79	78.69	−4.1
Linear SVC	91.8	95.65	3.85
Dummy classifier	95.62	92.02	−3.6
Linear discriminant analysis	90.21	93.7	3.49
Decision tree classifier	89.14	85.66	−3.48
SGD classifier	94.67	91.6	−3.08
AdaBoost classifier	72.49	69.57	−2.92
MLP classifier	93.99	91.18	−2.81
Gaussian NB	95.9	93.16	−2.74
Random forest classifier	92.53	95.22	2.68
Ridge classifier	95.11	92.44	−2.67
Calibrated classifier CV	93.88	92.02	−1.86
Extra trees classifier	94.08	92.61	−1.47
SVC	95.19	94.35	−0.85
Gradient boosting classifier	93.9	94.37	0.47
Logistic regression CV	95.4	95.22	−0.18
NuSVC	87.15	86.97	−0.17
Hist gradient boosting classifier	95.51	95.65	0.14
XGB classifier	95.66	95.52	−0.14

*Positive difference values = TP > TN, Negative = TN > TP*.

**Table 2:** True positive (TP), true negative (TN), and difference between tuned histogram-based gradient boosting (HGB), and XGBoost (XGB) performance (difference) on the validation set.

	**TP**			**TN**		
	**HGB**	**XGB**	**Difference**	**HGB**	**XGB**	**Difference**
Train	100	100	0	100	100	0
Validation	96.96	96.95	0.01	96.76	96.31	0.45
Test	94.60	94.67	−0.07	95.42	95.65	−0.23
Rest	0	0	0	96.18	96.83	−0.65
Hold Out	100	100	0	95.62	96.21	−0.59

*Positive difference values = HGB > XGB, Negative = XGB > HGB*.

### XGBoost Model (Classifier Performance Output)

Estimated TP, TN metrics, and the F1 score (F1) were calculated from labels estimated by the trained XGBoost against the video-detected ground truth labels (Table 3). Point estimate performance of the classifier was above 95% for all hit labeled impacts across all the data subsets (excluding the rest set where no TPs were present). Confidence intervals ranged from 92.51% for the test to 99.60% for the validation set. Point estimate true negative values ranged from slightly below 95% (94.54) for the hold out set to 98.65% for the validation set, while 95% CIs ranged from 91.49% (hold out set) to 100% (validation set). TP CIs suggest that there was no difference between validation and test sets, while performance on the hold out set was superior (not corrected). Overlapping CIs for TNs suggests no significant difference in classifier performance across datasets.

**Table 3:** Train, validation, test, rest, and holdout set number of impacts included and performance of classifier for true positives (TP) and true negatives (TN) as a percentage of correctly labeled data and F1 score (F1) with 95% confidence intervals (95% CI).

**Subset**	**Hits**	**Non Hits**	**TP (95% CI)**	**TN (95% CI)**	**F1 (95% CI)**
Train	1,106	1,106	100	100	1.00
			(100.00–100.00)	(100.00–100.00)	(1.00–1.00)
Validation	244	230	96.95	96.31	0.96
			(94.59–99.08)	(93.72–98.44)	(0.95–0.98)
Test	244	230	94.67	95.65	0.95
			(91.74–97.24)	(92.83–98.12)	(0.93–0.97)
Rest	0	10,257	0.00	96.83	0.00
				(96.49–97.16)	(0.00–0.00)
Holdout	57	238	100.00	96.21	0.92
			(100.00–100.00)	(93.62–98.37)	(0.87–0.97)

### Model Interpretation

**Figure 1:**
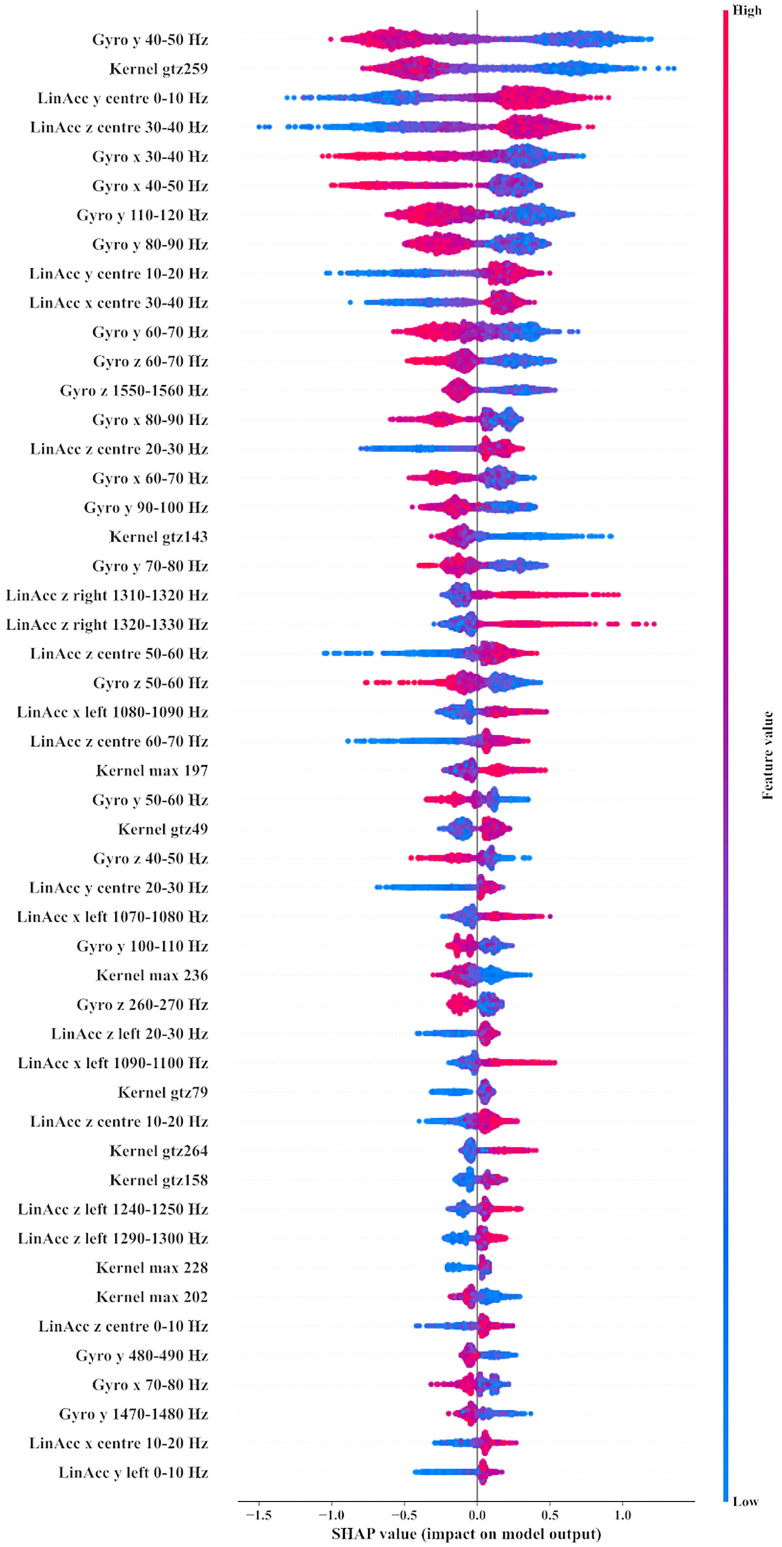
Top 50 features used by the XGBoost model. Gyro, Gyrometer; LinAcc, Linear accelerometer; x, X axis; y, Y axis; z, Z axis; Kernel, Convolutional kernel; max, Maximum value of kernel; ppv, Proportion of Positive Values in kernel. Values above 0 indicate contribution toward a positive label (hit), while values below 0 are contributions to a non-hit label.

Figure 1 shows the top 50 features used by the XGBoost model. Feature importance (*y* axis) goes from the most important (top) in descending order of importance. Each feature has individual impacts plotted from left to right, with color representing whether the value for that feature and observation was “high” (above the mean from memory, red) or “low,” lower than the mean (blue), with intensity as the distance. The *x* axis shows impact on the model. Values above 0 indicate contribution toward a positive label (hit), while values below 0 are contributions to a non-hit label. In Figure 1, it can be seen that the top 50 features were predominantly spectral in nature, with dominant frequency bands being under 150 Hz. Gyrometer and central linear accelerometer sensors were shown to contribute the majority of information to the classifier.

## Discussion

Our classification model that was developed using smart mouthguard technology and video verified impacts of elite Australian Rules Football players showed good performance, being able to correctly identify over 90% hits and non-hit captures. Additionally, we showed the importance of sub-150 Hz frequencies in developing the model from rotational and linear information.

A recent systematic review of the impact sensor literature (Patton et al., 2020) reported that the majority of eligible articles (64%) did not employ an observer or video verification for sensor-recorded events. This raises substantial concern that the head impact sensor literature may inaccurately identify impact events. Those articles that did not apply a verification method may be overestimating the head impact exposure by including false positive data (Patton et al., 2020). While 74% of eligible articles applied a filtering algorithm to automatically remove false positives, they did not also use an observer or video verification method to reaffirm false positives that were removed from the dataset (Patton et al., 2020). The sole use of a filtering algorithm has not been considered to be a valid replacement for observer and/or video verification of head impacts (Nevins et al., 2018), largely because the algorithms were not derived from on-field (i.e., game-play) data (Patton et al., 2020).

While most processing algorithms that are used to remove false positive and other spurious events remain proprietary, head impact telemetry system (Simbex, Lebanon, NH) has previously reported that their system compares the sensor-recorded kinematics to the expected acceleration signals for rigid body head acceleration (Crisco et al., 2004). Another method adopted by some authors in the field is to apply a threshold as a filter (e.g., removing all impacts <10 g peak linear acceleration and all impacts that surpass 200 g peak linear acceleration), (Rahimi and Recht, 2007; O'Connor et al., 2017). Optimal recording thresholds can only be determined *via* comprehensive video confirmation approaches using unfiltered data (Patton et al., 2020).

Video verification methods are not often used to establish reliability and validity of impacts. Wu et al. (2018) have previously reported on the training and validation of an impact classifier for an instrumented mouthguard in a small sample of seven collegiate football players and a total of 387 impacts collected from practice and games. The authors reported achieving 87.2% sensitivity and 93.2% precision and emphasized the importance of accurate impact detection. By way of comparison, the current study employed a similar methodology in a larger sample of male and female Australian Rules Football players with a larger dataset of 13,712 video verified body and head impacts, with the lowest sensitivity value of 94.67% (sensitivity = true positives/(true positives + false negatives)) and lowest precision of 94.42% (precision = true positives/(true positives + false positives)). The between-study differences in results can be related to multiple factors, including sample size, sports (helmeted vs. unhelmeted), technology, and statistical methodology.

A second study to report results of a mouthguard-based classifier by Kieffer et al. (2020) utilized head injury metrics as features with support vector machine and an artificial neural network as their base algorithms (Benzel et al., 2016). Performance of their impact classifier was evaluated using positive predictive value (otherwise known as precision) when players were both on and off the field (combined) and on field from non-helmeted Rugby players. Combined PPV was 91.2% while on field alone was 96.4%. The presented classifier showed PPV ranging from 94.42% (test) to 100% (holdout) with both on and off field data.

Machine-learning-based models have been criticized for their opaque nature compared with traditional statistical modeling methods (e.g., decision trees, logistic regression) (Lundberg et al., 2020). Exploration of feature importance using SHAP showed low frequency (<150 Hz) rotational power spectra density features to assist in helping model performance. Wu et al. (2018) showed their support vector machine based classifier to be utilizing low frequency components; however, they reported most of the importance to be in linear accelerometer derived features, at much lower frequencies (<30 Hz). This difference in reported feature importance may be due to several factors, including the classifier method used, different feature types used, and potentially different impact characteristics of helmeted compared with non-helmeted sports. In this study, we used a randomized tree based boosted method (XGBoost), which makes inherent use of interactions within the data and generates a multidimensional decision boundary in a stepwise fashion. Conversely, Wu et al. (2018) used a radial kernel support vector machine that attempts to find a linear decision boundary between classes when the features have been projected to a higher dimensional space. This tends to produce a smoother decision boundary (Cortes and Vapnik, 1995). Additionally, Wu et al. (2018) utilized several different groups of features, including power spectra density and wavelet-transformed time frequency information, time domain peak information, and biomechanical-based features. In comparison, our classifier used power spectra density based features and randomized kernels that can be used to examine both frequency and shape-based characteristics of the signal (Dempster et al., 2020).

Finally, Wu et al. (2018) developed a classifier for use in American Football where players wear protective helmets to guard against head injuries, while the presented classifier was produced using AFL data, in which helmets are not worn. There may be important differences in how impacts are represented in the frequency domain between helmeted and non-helmeted sports, with the helmet absorbing more of the higher frequency components and allowing only sub 50 Hz based kinetic energy to be transferred into the head.

### Limitations

This study has several limitations. While video review hits and non-hit captures were noted by the video reviewers, there is a possibility that hits that were not of sufficient magnitude to reach the mouth guard's 10 g threshold and were therefore not captured during the process could have been present but not noted. We did not attempt to identify these impacts during video review. Although, our classifier showed high performance in distinguishing between hits and non-hits, this performance may only reflect impacts at 10 gs or greater. The classifier was also applied specifically to the Nexus A9 mouthguard for classifying the impact of male and female Australian Rules Football players. Whether a similar performance level is achievable for other contact sports using this classifier is unknown. The classifier was applied to adult athletes and may not generalize to child and adolescent athletes. Additionally, the classifier was applied to elite level players and may not generalize to amateur and community level athletes. Training of the classifier was accomplished using data from four female and sixty male Australian Rules Football players. Bias in machine learning/artificial intelligence methods of classification are widely known. The lack of recorded data from women in the training set may lead to inaccurate results when attempting to classify impacts in professional women athletes. Although standardization of the time series during processing may remove bias that is present due to magnitude differences between the sexes based on body mass, other potential sources of bias, such as differences in impact behavior, cannot be ruled out. Finally, the classifier did not include data from all possible positions in Australian Rules Football. Because different positions have different impact probabilities and the form of impact may vary, it is possible that some types of impact may not have been recorded.

### Practical Implications

Video verification is an essential element for collecting reliable kinematic data from sensors. Establishing a method of synchronization between video footage and the output from the sensors is critical to the video verification process. An algorithm-driven classifier, in conjunction with the video verification method, optimizes the integrity of the data. Validating a classifier that applies these two principals improves the reliability of the data for making decisions around the suitability of an athlete to remain in play, or to be removed for a medical assessment, following a hard blow to the head. Additionally, this study showed the importance of frequency ranges <150 Hz in creating a tree-based model to accurately identify impacts. Both linear and rotational spectral information provided the majority of information to develop the classifier as evidenced by SHAP, suggesting that temporally based metrics (e.g., peak values) may not be required.

## Conclusions

It is essential for a valid verification method to be used to confirm sensor-recorded events and to remove false positives. Video verification in combination with an algorithm-driven classifier can provide an accurate method for filtering data and optimizing the integrity of the dataset. The current study showed that the classifier for the Nexus A9 mouthguard is an accurate system for identifying impacts to the body and head in elite level Australian Rules Football players. Future research should focus on further validation of impact sensor classifiers in other contact and collisions sports, and also across the various levels of sport.

## Data Availability Statement

The datasets presented in this article are not readily available because of contractual obligations surrounding privacy of data as well as the need to protect intellectual property. Consideration will be made for access requests to data not implicated by these obligations. Requests to access the datasets should be directed to peter@hitiq.co.

## Ethics Statement

Ethical review and approval was not required for the study on human participants in accordance with the local legislation and institutional requirements. The patients/participants provided their written informed consent to participate in this study.

## Author Contributions

PG conceptualized the study, wrote the first draft of the manuscript, and performed the statistical analyses. PG, BN, and SA conceptualized the statistical analyses. All authors critically reviewed the manuscript. All authors read and approved the last version of this manuscript.

## Funding

Funding for this study was provided by HitIQ Limited.

## Conflict of Interest

HitIQ Ltd. is a company with commercial and proprietary interest in the mouthguard and classifier used in this study. PG, ND, BN, and SA are employed by HitIQ Ltd. AG serves as a scientific advisor for HitIQ Ltd. BN and SA are former employees of HitIQ Ltd. AG has a clinical practice. He has been a consultant to Rugby Australia ltd. He has received travel funding from professional sporting bodies and commercial organizations meetings, scientific conferences, workshops, and symposiums. He has received research funding from the National Rugby League (NRL) for the Retired Players Brain Health research program. Grant Iverson serves as a scientific advisor for NanoDxTM (formerly BioDirection, Inc.), Sway Operations, LLC, and Highmark, Inc. He has a clinical and consulting practice. He has received research funding from several test publishing companies, including ImPACT Applications, Inc., CNS Vital Signs, and Psychological Assessment Resources (PAR, Inc.). He has received research funding as a principal investigator from the National Football League. He acknowledges unrestricted philanthropic support from ImPACT Applications, Inc. and the National Rugby League. These entities were not involved in the study design, collection, analysis, interpretation of data, the writing of this article, or the decision to submit it for publication. The remaining authors declare that the research was conducted in the absence of any commercial or financial relationships that could be construed as a potential conflict of interest.

## Publisher's Note

All claims expressed in this article are solely those of the authors and do not necessarily represent those of their affiliated organizations, or those of the publisher, the editors and the reviewers. Any product that may be evaluated in this article, or claim that may be made by its manufacturer, is not guaranteed or endorsed by the publisher.

## References

[B1] Abraham A. Pedregosa F. Eickenberg M. Gervais P. Mueller A. Kossaifi J.. (2014). Machine learning for neuroimaging with scikit-learn. Front. Neuroinform. 8:14. 10.3389/fninf.2014.00014 24600388 PMC3930868

[B2] Bailey A. M. Sherwood C. P. Funk J. R. Crandall J. R. Carter N. Hessel D.. (2020). Characterization of concussive events in professional American football using videogrammetry. Ann. Biomed. Eng. 48, 2678–2690. 10.1007/s10439-020-02637-3 33025319

[B3] Baugh C. M. Stamm J. M. Riley D. O. Gavett B. E. Shenton M. E. Lin A.. (2012). Chronic traumatic encephalopathy: neurodegeneration following repetitive concussive and subconcussive brain trauma. Brain Imag. Behav. 6, 244–254. 10.1007/s11682-012-9164-5 22552850

[B4] Bazarian J. J. Zhu T. Zhong J. Janigro D. Rozen E. Roberts A.. (2014). Persistent, long-term cerebral white matter changes after sports-related repetitive head impacts. PLoS ONE 9:e94734. 10.1371/journal.pone.0094734 24740265 PMC3989251

[B5] Benzel E. C. Miele V. J. Bartsch A. J. C. (2016). Classification of Impacts from Sensor Data. US Patent 9,289,176.

[B6] Carey L. Stanwell P. Terry D. P. Mcintosh A. S. Caswell S. V. Iverson G. L.. (2019). Verifying head impacts recorded by a wearable sensor using video footage in rugby league: a preliminary study. Sport Med. Open 5:9. 10.1186/s40798-019-0182-3 30874938 PMC6419663

[B7] Caswell S. V. Lincoln A. E. Stone H. Kelshaw P. Putukian M. Hepburn L.. (2017). Characterizing verified head impacts in high school girls' lacrosse. Am. J. Sports Med. 45, 3374–3381. 10.1177/0363546517724754 28918649

[B8] Chen T. Guestrin C. (2016). XGBoost: a scalable tree boosting system, in KDD'16: Proceedings of the 22nd ACM SIGKDD International Conference on Knowledge Discovery and Data Mining (San Francisco, CA), 785–794. 10.1145./2939672.2939785

[B9] Cortes C. Vapnik V. (1995). Support-vector networks. Mach. Learn. 20, 273–297. 10.1007/BF00994018

[B10] Cortes N. Lincoln A. E. Myer G. D. Hepburn L. Higgins M. Putukian M.. (2017). Video analysis verification of head impact events measured by wearable sensors. Am. J. Sports Med. 45, 2379–2387. 10.1177/0363546517706703 28541813

[B11] Crisco J. J. Chu J. J. Grenwald R. M. A. (2004). An algorithm for estimating acceleration magnitude and impact location using multiple non-orthogonal single-axis accelerometers. J. Biomech. Eng. 126, 849–854. 10.1115/1.1824135 15796345

[B12] Dai J. B. Li A. Y. Haider S. F. Tomaselli R. Gometz A. Sobotka S.. (2018). Effects of game characteristics and player positions on concussion incidence and severity in professional football. Orthop. J. Sport Med. 6:2325967118815448. 10.1177/2325967118815448 30627588 PMC6311573

[B13] Davis G. A. Makdissi M. Bloomfield P. Clifton P. Echemendia R. Falvey E.. (2019b). International consensus definitions of video signs of concussion in professional sports. Br. J. Sports Med. 53, 1264–1267. 10.1136/bjsports-2019-100628 30954947

[B14] Davis G. A. Makdissi M. Bloomfield P. Clifton P. Echemendia R. J. Falvey É. C.. (2019a). International study of video review of concussion in professional sports. Br. J. Sports Med. 53, 1299–1304. 10.1136/bjsports-2018-099727 30262454

[B15] Dempster A. Petitjean F. Webb G. I. (2020). OCKET: exceptionally fast and accurate time series classification using random convolutional kernels. Data Min. Knowl. Discov. 34, 1454–1495. 10.1007/s10618-020-00701-z

[B16] Donaldson L. Asbridge M. Cusimano M. D. B. (2013). Bodychecking rules and concussion in elite hockey. PLoS ONE 8:e69122. 10.1371/journal.pone.0069122 23874888 PMC3714233

[B17] Eckner J. T. Sabin M. Kutcher J. S. Broglio S. P. N. (2011). No evidence for a cumulative impact effect on concussion injury threshold. J. Neurotrauma 28, 2079–2090. 10.1089/neu.2011.1910 21815783 PMC4346375

[B18] Ellenbogen R. G. Batjer H. Cardenas J. Berger M. Bailes J. Pieroth E.. (2018). National football league head, neck and spine committee's concussion diagnosis and management protocol: 2017–18 season. Br. J. Sports Med. 52. 903-904. 10.1136/bjsports-2018-099203 29549147

[B19] Gabler L. F. Huddleston S. H. Dau N. Z. Lessley D. J. Arbogast K. B. Thompson X.. (2020). On-field performance of an instrumented mouthguard for detecting head impacts in American football. Ann. Biomed. Eng. 48, 2599–2612. 10.1007/s10439-020-02654-2 33078368

[B20] Gardner A. J. Iverson G. L. Levi C. R. Schofield P. W. Kay-Lambkin F. Kohler R. M. N.. (2014a). A systematic review of concussion in rugby league. Br. J. Sports Med. 49, 495–498. 10.1136/bjsports-2013-093102 24723636

[B21] Gardner A. J. Iverson G. L. Williams W. H. Baker S. Stanwell P. A. S. (2014b). A systematic review and meta-analysis of concussion in rugby union. Sport Med. 44, 1717–1731. 10.1007/s40279-014-0233-3 25138311

[B22] Gardner A. J. Kohler R. McDonald W. Fuller G. W. Tucker R. Makdissi M. (2018). The use of sideline video review to facilitate management decisions following head trauma in super rugby. Sport Med. Open 4:20. 10.1186/s40798-018-0133-4 29797099 PMC5968014

[B23] Gavett B. E. Stern R. A. McKee A. C. (2011). Chronic traumatic encephalopathy: a potential late effect of sport-related concussive and subconcussive head trauma. Clin. Sports Med. 30, 179–88. 10.1016/j.csm.2010.09.007 21074091 PMC2995699

[B24] Gysland S. M. Mihalik J. P. Register-Mihalik J. K. Trulock S. C. Shields E. W. Guskiewicz K. M. (2011). The relationship between subconcussive impacts and concussion history on clinical measures of neurologic function in collegiate football players. Ann. Biomed. Eng. 40, 14–22. 10.1007/s10439-011-0421-3 21994067

[B25] Kieffer E. E. Begonia M. T. Tyson A. M. Rowson S. A. T. (2020). Two-phased approach to quantifying head impact sensor accuracy: in-laboratory and on-field assessments. Ann. Biomed. Eng. 48, 2613–2625. 10.1007/s10439-020-02647-1 33051745

[B26] King D. Hume P. Gissane C. Brughelli M. Clark T. (2016). The influence of head impact threshold for reporting data in contact and collision sports: systematic review and original data analysis. Sport Med. 46, 151–169. 10.1007/s40279-015-0423-7 26545363

[B27] Lundberg S. M. Erion G. Chen H. DeGrave, A. Prutkin J. M. Nair B. Katz R. Himmelfarb J. Bansal N. Lee S.-I. (2020). From local explanations to global understanding with explainable AI for trees. Nat. Mach. Intell. 2, 56–67. 10.1038/s42256-019-0138-9 32607472 PMC7326367

[B28] Lundberg S. M. Lee S.-I. (2017). A unified approach to interpreting model predictions, in Proceedings of the 31st Conference on Neural Information Processing Systems (NIPS 2017), eds Guyon I. Luxburg U. V Bengio S. Wallach H. Fergus R. Vishwanathan S. Garnett R. (Long Beach, CA).

[B29] Makdissi M. Davis G. (2016). The reliability and validity of video analysis for the assessment of the clinical signs of concussion in Australian football. J. Sci. Med. Sport 19, 859–863. 10.1016/j.jsams.2016.02.015 27009774

[B30] McCrea M. Iverson G. L. Echemendia R. J. Makdissi M. Raftery M. (2013). Day of injury assessment of sport-related concussion. Br. J. Sports Med. 47, 272–284. 10.1136/bjsports-2013-092145 23479484

[B31] McCrory P. Meeuwisse W. Dvorak J. Aubry M. Bailes J. Broglio S.. (2017). Consensus statement on concussion in sport, in Proceeding of the 5th International Conference on Concussion in Sport (Berlin).

[B32] Merchant-Borna K. Asselin P. Narayan D. Abar B. Jones C. M. C. Bazarian J. J. N. (2016). Novel method of weighting cumulative helmet impacts improves correlation with brain white matter changes after one football season of sub-concussive head blows. Ann. Biomed. Eng. 44, 3679–3692. 10.1007/s10439-016-1680-9 27350072

[B33] Montenigro P. H. Alosco M. L. Martin B. M. Daneshvar D. H. Mez J. Chaisson C. E.. (2017). Exposure predicts later-life depression, apathy, executive dysfunction, and cognitive impairment in former high school and college football players. J. Neurotrauma 34, 328–340. 10.1089/neu.2016.4413 27029716 PMC5220530

[B34] Nevins D. D. Hildenbrand K. Kensrud J. Vasavada A. Smith L. (2018). Laboratory and field evaluation of a small form factor head impact sensor in unhelmeted play. J. Sport Eng. Technol. 232, 242–254. 10.1177/1754337117739458

[B35] O'Connor K. L. Peeters T. Szymanski S. Broglio S. P. I. (2017). Individual impact magnitude vs. cumulative magnitude for estimating concussion odds. Ann. Biomech. Eng. 45, 1985–1992. 10.1007/s10439-017-1843-3 28455786

[B36] Oliphant T. E. P. (2007). Python for scientific computing. Comput. Sci. Eng. 9, 10–20. 10.1109/MCSE.2007.5827295638

[B37] Patton D. A. Huber C. M. Jain D. Myers R. K. McDonald C. C. Margulies S. S.. (2020). Head impact sensor studies in sports: a systematic review of exposure confirmation methods. Ann. Biomed. Eng. 48, 2497–2507. 10.1007/s10439-020-02642-6 33051746 PMC7674240

[B38] Press J. N. Rowson S. (2016). Quantifying head impact exposure in collegiate women's soccer. Clin. J. Sport Med. 27, 104–110. 10.1097/JSM.0000000000000313 26978008

[B39] Rahimi A. Recht B. (2007). Random features for large-scale kernel machines, in NIPS.

[B40] Ramkumar P. N. Navarro S. M. Haeberle H. S. Luu B. C. Jang A. Frangiamore S. J.. (2019). Concussion in American vs. European professional soccer: a decade-long comparative analysis of incidence, return to play, performance, and longevity. Am. J. Sports Med. 47, 2287–2293. 10.1177/0363546519859542 31303010

[B41] Rubin L. H. Tierney R. Kawata K. Wesley L. Lee J. H. Blennow K.. (2019). NFL blood levels are moderated by subconcussive impacts in a cohort of college football players. Brain Inj. 33, 456–462. 10.1080/02699052.2019.1565895 30776989

[B42] Stemper B. D. Shah A. S. Harezlak J. Rowson S. Mihalik J. P. Duma S. M.. (2018). Comparison of head impact exposure between concussed football athletes and matched controls: evidence for a possible second mechanism of sport-related concussion. Ann. Biomed. Eng. 47, 2057–2072. 10.1007/s10439-018-02136-6 30362082 PMC6785644

[B43] Stitt D. Draper N. Alexander K. Kabaliuk N. (2021). Laboratory validation of instrumented mouthguard for use in sport. Sensors 21:6028. 10.3390/s21186028 34577235 PMC8472105

[B44] Welch P. (1967). The use of fast Fourier transform for the estimation of power spectra: a method based on time averaging over short, modified periodograms. IEEE Trans. Audio Electroacoust. 15, 70–73. 10.1109/TAU.1967.116190127295638

[B45] Wu L. C. Kuo C. Loza J. Kurt M. Laksari K. Yanez L. Z.. (2018). Detection of American football head impacts using biomechanical features and support vector machine classification. Sci. Rep. 8, 1–14. 10.1038/s41598-017-17864-3 29321637 PMC5762632

